# Genetic polymorphisms as non-modifiable susceptibility factors to laryngeal cancer

**DOI:** 10.1042/BSR20191188

**Published:** 2020-05-05

**Authors:** Paula Escalante, Tamara Barría, Miguel Cancino, Maritza Rahal, Leslie Cerpa, Christopher Sandoval, Sebastian Molina-Mellico, Marcelo Suárez, Matias Martínez, Dante Daniel Cáceres, Luis Abel Quiñones, Nelson Miguel Varela

**Affiliations:** 1 Laboratory of Chemical Carcinogenesis and Pharmacogenetics, Department of Basic-Clinical Oncology, Faculty of Medicine, University of Chile, Santiago, Chile & Latin American Network for the Implementation and Validation of Clinical Pharmacogenomics Guidelines (RELIVAF-CYTED), Madrid, Spain; 2 Otorhinolaryngology Service, Barros Luco Hospital, Santiago, Chile; 3 School of Public Health, Faculty of Medicine, University of Chile, Santiago, Chile; 4 Health Science Faculty, Tarapacá University, Iquique, Chile

**Keywords:** Biomarkers, EGF, EGFR, Genomics, Laryngeal cancer, Polymorphism, PTGS2, TP53

## Abstract

Laryngeal squamous cell carcinoma (LSCC) is a highly disabling disease to the patient, affecting speech, swallowing and respiratory skills. Smoking and alcohol abuse are principal risk factors linked to this disease. Genetic factors can be involved in carcinogenesis by controlling the cell cycle, cell survival, angiogenesis, and invasiveness. Single nucleotide polymorphisms (SNPs) involving specific genes could modulate the risk of LSCC related to known carcinogens by modifying cellular responses, but not all genetic associations are known.

In a case–control study, we assess the associations between cyclooxygenase-2 (COX2), epidermal growth factor (EGF), EGF receptor (EGFR), and tumor suppressor P53 SNPs on the risk of LSCC development in the Chilean population. A total of 85 LSCC patients and 95 healthy volunteers were recruited. SNPs genotype were analyzed from genomic DNA by Polymerase Chain Reaction (PCR)-Restriction Fragment Length Polymorphism (RFLP) and associations were estimated by odds ratios (ORs) using unconditional logistic regressions.

A significant association between COX2 and TP53 SNP and LSCC risk was found, with an OR = 3.27 for COX2 c.-1329A>G (rs689466) SNP, and an OR = 1.94 for TP53 c.215C>G, Pro72Arg (rs1042522) SNP. These findings suggest that COX2 c.-1329A>G and TP53 c.215C>G (Pro72Arg) SNPs may be risk factors for LSCC.

Through this research, we identify two low penetrance genetic variants that may be evaluated as novel biomarkers for this disease, in South American Mestizo populations.

## Introduction

Laryngeal squamous cell carcinoma (LSCC) is one of the different types of head and neck squamous carcinoma, affects most frequently in people aged over 65 and is approxiamtely three to ten times more frequent in males than females [1,2]. Tobacco and alcohol abuse are the principal risk factors of LSCC, and combined consumption has a multiplicative effect in the relative risk of cancer [3,4]. Carcinogens in cigarette smoke are activated by xenobiotic metabolizing enzymes during the detoxification process, producing highly reactive intermediates which can damage exposed cells, and form DNA adducts, and therefore can lead to cancer if it causes tumor suppressor loss-of-function or oncogene activation [3,5,6].

Inflammation signaling pathways, mitogen signaling, tumor suppressor genes, and xenobiotic metabolizing enzymes are factors that can be involved in carcinogenesis and malignant transformation, by controlling the cell cycle, cell survival, angiogenesis, and invasiveness. Genetic variants as, for example, single nucleotide polymorphisms (SNPs) could play an important role in carcinogenesis, by disrupting the normal function of proteins encoded by genes involved in these cellular processes [2,7,8]. These SNPs are usually low penetrance risk factors with small impact in overall relative risk of cancer, but combined with external risk factors like cigarette smoking and alcohol consumption, could exert a synergistic effect, hence exposing a specific population to a higher probability of developing cancer.

Several pathways can be involved in LSCC risk and pathogenesis. Prostaglandin-endoperoxide synthase 2 (PTGS2) or Cyclooxygenase-2 (COX2) is an enzyme involved in inflammation and tissue damage responses, which could lead to cancer. Two SNPs rs20417 (c.-899G>C, C allele) and rs689466 (c.-1329A>G, A allele) increasing COX2 protein expression have been reported as potential susceptibility biomarkers [9–14]. On the other hand, epidermal growth factor receptor (EGFR), a tyrosine kinase receptor activated by the epidermal growth factor (EGF), is essential in laryngeal squamous epithelium differentiation, survival, and proliferation. Thus, increased signaling on this pathway has been linked to several types of cancer [15–17]. EGF rs4444903 SNP (c.-382A>G, A allele) is associated with increased EGF expression, and EGFR rs2227983 SNP (c.1562G>A, A allele) produce an aminoacidic change from Arginine to Lysine (R521K) in this receptor associated with decreased EGF affinity and therefore, lower tyrosine kinase signaling [18–21]. Finally, *TP53*, a gene coding for a key tumor suppressor protein, where the G allele of the rs1042522 (c.215C>G; codon Pro72Arg), has been associated with enhanced tumor suppressor activity through increased pro-apoptotic activity [17,22–26].

These SNPs have been previously associated with different types of cancer, but the evidence is inconclusive and sometimes, contradictory. Therefore, in the present study, we aimed to identify the impact of these SNPs alone or combined with cigarette smoking and/or alcohol consumption in the risk of LSCC, in a population from the city of Santiago de Chile.

## Methods

### Design and study populations

A case–control study was performed, where 85 LSCC patients with previous histological diagnosis confirmation and 95 confirmed cancer-free volunteers were recruited from March 2012 to December 2016. Blood samples were collected from the Barros Luco Trudeau Hospital and the Biobank from the Laboratory of Chemical Carcinogenesis and Pharmacogenetics (CQF) at the Faculty of Medicine of the University of Chile. The present study was approved by the Ethics Committee for Human Research at the Faculty of Medicine of the University of Chile (January 2012) and was performed following the Declaration of Helsinki and Good Clinical Practices (GCP). All the patients included in the present study underwent an informed consent process, and signed an informed consent document approved by the ethics committee.

### SNP genotyping

Genomic DNA was extracted from peripheral blood lymphocytes using commercial kit (E.Z.N.A.® Blood DNA Mini Kit, Omega Bio-tek, U.S.A.). Genotype was determined by Polymerase Chain Reaction (PCR)-based Restriction Fragment Length Polymorphism (RFLP) [13,27–29]. PCRs were performed in a G-Storm Thermocycler, model GS00482, using 0.2 nmol of each Primer (IDT Fermelo-Biotec, Chile), 2 μl of Genomic DNA stock solution, and 1× PCR Master Mix (MyTaq DNA Polymerase, Bioline, U.K.), following the manufacturer-recommended protocol for PCRs. Primer pairs, PCR product sizes, and restriction enzymes used for determination of each genotype are summarized in Table 1. DNA fragments length were visualized by electrophoresis in 2% agarose gel or 18% polyacrylamide gel depending on the fragment lengths and revealed with ethidium bromide. Supplementary Figure S1 shows representatives results of the PCR-RFLP for each SNP.

**Table 1:** Summary of primers and restriction enzymes for SNP genotyping

SNP	Forward primer (5′–3′)	Reverse primer (5′–3′)	Amplicon size	Restriction enzyme	Reference
COX-2 -765 C>G	AGGCAGGAAACTTTATATTGG	ATGTTTTAGTGACGACGCTTA	309	*SsiI*	Shin et al*.* (2012) [13]
COX-2 -1195 A>G	CCCTGAGCACTACCCATGAT	GCCCTTCATAGGAGATACTGG	273	*PvuII*	Shin et al*.* (2012) [13]
EGF +61 A>G	TGTCACTAAAGGAAAGGAGGT	TTCACAGAGTTTAACAGCCC	242	*AluI*	Shahbazi et al*.* (2002) [27]
EGFR R521K G>A	TGCTGTGACCCACTCTGTCT	CCAGAAGGTTGCACTTGTCC	155	*MvaI*	Sasaki et al. (2009) [28]
TP53 P72R C>G	AATGGATGATTTGATGCTGTCCC	CGTGCAAGTCACAGACTTGGC	259	*BstUI*	Ueda et al. (2005) [29]

To validate the specificity of the primer pairs to the target DNA gene sequence, alignment of each primer with the *Homo sapiens* genome assembly was performed using the bioinformatics software tools Primer Blast (NCBI, GRCh38.p12) and *In-Silico* PCR (UCSC Genome Browser, GRCh38.hg38) to ensure that no unspecific PCR products were obtained in the established cycling conditions. Restriction sites were manually checked for predicted PCR products considering each possible allele and compared with the results observed in the reference literature to verify that the expected restriction fragments were consistent with previously published data, and later compared with the PCR-RFLP products obtained in our laboratory. Samples were genotyped in duplicated and 20% of the samples were re-analyzed to ensure the reproducibility of our results.

### Statistical analysis

Sample size was calculated using Open Epi 3.01, based on the frequencies of TP53 c.215C>G (P72R) SNP observed for squamous skin carcinoma by Loeb et al*.* (2012) [26], with a proportion of cases exposed of 61%, and controls exposed of 41%, considering a two-sided confidence level of 95%, detection power of 80%, setting a minimum sample size of 82 subjects per group.

Risk alleles were assigned as follows: C allele for COX2 c.-899G>C, and A allele for c.-1329A>G, both associated with increased COX2 protein expression [12], A allele for EGF c.-382A>G SNP, associated with lower *in-vitro* expression of EGF protein compared with the G allele [18], G allele for EGFR c.1562G>A (R521K), associated with normal receptor activity, and higher relative to the missense substitution in the A allele of EGFR [20], and finally the C allele for TP53 SNP c.215C>G, associated with reduced pro-apoptotic activity of P53 protein (P72R) [22]. LSCC risk was evaluated using crude and adjusted odds ratio (AOR) adjusted by confounding factors (smoke, alcohol consumption, and gender) through logistic unconditional regression. To evaluate gene–environment interaction, we calculated the Interaction Odds Ratio (IOR) using an extension of two-by-four table, through logistic regression with interacting terms. In the present study, we assumed independence between the genotypes and the exposure to environmental risk factors and defined a significant gene–environment interaction if the IOR was higher than 2 [30,31]. Statistical analyses were performed using Stata 13.0 statistical software. A *P*-value of less than 0.05 was considered statistically significant.

## Results

### Study population

Clinical characteristics and genotypes of the enrolled subjects are summarized in Table 2. There was no difference in mean age and male-to-female ratio between cases and controls, but the frequency of cigarette smoking habit was statistically different between both groups (*P*<0.001), and proportion of alcohol consumption habit was higher in cases compared with controls, although it was not statistically significant. As expected, cigarette consumption was highly associated with LSCC risk (smoking OR: 5.22, 95% CI: 2.74–9.96, *P*<0.001). All SNP genotypes were detectable in both groups and genotypic frequencies in control samples were in Hardy–Weinberg equilibrium.

**Table 2:** General characteristics and genotypic frequencies of COX-2 -765 G>C, COX-2 -1195 A>G, EGF rs4444903, EGFR rs2227983, and P53 P72R SNPs of the studied population

	Cases, *n* (%)	Controls, *n* (%)	OR	CI	*P*-value	Adjusted OR*	CI	*P*-value
***n***	85	95						
**Age, mean (SD)**	64.49 (11.09)	62.74 (12.49)			0.338			
**Male**	78 (91.8)	88 (92.6)			0.523			
**Female**	7 (8.2)	7 (7.4)						
Non-smokers	21 (24.7)	60 (63.2)	1.0	Reference				
**Smokers**	**64 (75.3)**	**35 (36.8)**	**5.22**	**2.74–9.96**	**<0.001**			
**Non-alcohol consumers**	53 (62.4)	72 (75.8)	1.0	Reference				
**Alcohol consumers, *n (%)***	32 (37.6)	23 (24.2)	1.89	0.99–3.59	0.052			
**Disease stage**								
**Stage I**	17 (20.0)							
**Stage II**	17 (20.0)							
**Stage III**	13 (15.3)							
**Stage IV**	31 (36.5)							
**Unknown**	7 (8.2)							
**COX-2 -765 C>G (rs20417)**								
G/G	50 (58.8)	64 (67.4)	1.0	Reference		1.0	Reference	
G/C	32 (37.6)	30 (31.6)	1.365	0.734–2.539	0.325	1.673	0.838–3.338	0.144
C/C	3 (3.5)	1 (1.1)	3.840	0.388–38.04	0.250	5.934	0.477–73.86	0.166
C/C + C/G	35 (41.2)	31 (32.6)	1.445	0.786–2.656	0.236	1.783	0.904–3.518	0.095
G/C + G/G	82 (96.5)	94 (98.9)	1.0	Reference		1.0	Reference	
C/C	3 (3.5)	1 (1.1)	3.439	0.351–33.71	0.289	4.850	0.40–58.83	0.215
**COX-2 -1195 A/G (rs689466)**								
G/G	11 (12.9)	9 (9.5)	1.0	Reference		1.0	Reference	
**A/G**	**20 (23.5)**	**50 (52.6)**	**0.327**	**0.118–0.910**	**0.032**	**0.252**	**0.080–0.793**	**0.018**
A/A	54 (63.5)	36 (37.9)	1.227	0.462–3.259	0.681	1.167	0.395–3.450	0.78
A/A + G/A	74	59 (62.1)	0.704	0.277–1.792	0.462	0.629	0.225–1.757	0.376
G/A + G/G	31	86 (90.5	1.0	Reference		1.0	Reference	
A/A*	**54 (63.5)**	**36 (37.9)**	**2.855**	**1.558–5.232**	**0.001**	**3.273**	**1.665–6.435**	**0.001**
**EGF +61 A>G (rs4444903)**								
G/G	20 (23.5)	31 (32.6)	1.0	Reference		1.0	Reference	
G/A	45 (52.9)	50 (52.6)	1.395	0.699–2.785	0.345	1.554	0.725–3.328	0.257
A/A	20 (23.5)	14 (14.7)	2.214	0.914–5.363	0.078	2.313	0.876–6.110	0.091
A/A + G/A	65 (76.5)	64 (67.4)	1.574	0.814–3.045	0.178	1.728	0.836–3.572	0.140
G/A + G/G	65 (76.5)	81 (85.3)	1.0	Reference		1.0	Reference	
A/A	20 (23.5)	14 (14.7)	1.780	0.835–3.795	0.135	1.735	0.757–3.975	0.193
**EGFR R521K G>A (rs2227983)**								
A/A	6 (7.1)	8 (8.4)	1.0	Reference		1.0	Reference	
G/A	37 (43.5)	33 (34.7)	1.495	0.470–4.759	0.496	2.073	0.586–7.332	0.258
G/G	42 (49.4)	54 (56.8)	1.037	0.260–2.162	0.594	1.425	0.417–4.871	0.573
G/G + G/A	79 (92.9)	87 (91.6)	1.211	0.402–3.642	0.734	1.666	0.503–5.520	0.403
G/A + A/A	43 (50.6)	41 (43.2)	1.0	Reference		1.0	Reference	
G/G	42 (49.4)	54 (56.8)	0.742	0.412–1.335	0.319	0.779	0.409–1.485	0.448
**Tumor suppressor P53 P72R C>G (rs1042522)**								
G/G	44 (51.8)	64 (67.4)	1.0	Reference		1.0	Reference	
G/C	39 (45.9)	28 (29.5)	2.026	1.091–3.762	0.025	2.038	1.038–4.000	0.039
C/C	2 (2.4)	3 (3.2)	0.970	0.156–6.044	0.974	1.006	0.139–7.273	0.995
C/C + C/G*	**41 (48.2)**	**31 (32.6)**	**1.924**	**1.051–3.520**	**0.034**	**1.939**	**1.003–3.745**	**0.049**
G/C + G/G	83 (97.6)	92 (96.8)	1.0	Reference		1.0	Reference	
C/C	2 (2.4)	3 (3.2)	0.740	0.120–4.532	0.496	0.765	0.108–5.437	0.789

Risk of LSCC is expressed as OR (Crude OR) and AOR, adjusted by cigarette smoking, alcohol consumption, and sex. (*) Indicates a statistically significant association between risk factor and LSCC OR.

### LSCC risk association with SNP genotype

We calculated the corresponding OR for LSCC risk according to COX2 c.-899G>C, COX2 c.-1329A>G, EGF c.-382A>G, EGFR c.1562G>A (R521K), and TP53 c.215C>G SNPs genotype, adjusted by cigarette smoking, alcohol consumption and gender, and considering different penetrance models (dominant, recessive, and additive) for each SNP (Table 2). Two genotypes were associated with increased LSCC risk: COX2 c.-1329A/A compared with A/G plus G/G genotypes (recessive penetrance, AOR: 3.27, 95% CI: 1.665–6.435, *P*-value: 0.001), and TP53 c.215C/C plus C/G genotypes compared with G/G genotype (dominant penetrance, AOR: 1.94, 95% CI: 1.003–3.745, *P*-value: 0.049). Inversely, the heterozygous genotype A/G of the COX2 c.-1329 SNP had a protector association with LSCC risk (additive penetrance, AOR: 0.25, 95% CI: 0.080–0.793, *P*-value: 0.018). These results suggest a direct association between the genes and risk of LSCC for COX2 c.-1329 (rs689466) and TP53 c.215 (rs1042522) SNPs.

### Combined effect of SNP genotype and smoking habit in LSCC risk

To assess the gene–environment interaction and its effect on laryngeal cancer risk, we used a two-by-four table to determine the stratified ORs for the risk genotype, the environmental risk factor, and for the combined gene–environment interaction. With these data, we calculated the IOR for the joint effect of the combined risk factors to determine if there is a significant synergic relation between the individual SNPs and the environmental factor, in this case, the effect of cigarette smoking, which would explain how the individual SNPs affect the cigarette- associated LSCC risk. Considering the tendencies observed in the results described in Table 2 we applied a dominant penetrance model for the COX2 c.-899G>C, EGF c.-382A>G and TP53 c.215C>G (P72R) SNPs, and for the COX2 c.-1329 A>G and EGFR c.1562G>A (R521K) SNPs we applied a recessive model for risk analysis (Table 3).

**Table 3:** LSCC risk for combinations of cigarette smoking habit and COX-2 -765 G>C, COX-2 -1195 A>G, EGF+61 A>G, EGFR R521K G>A, and P53 P72R SNPs genotypes

Genotype	Smoking	Cases	Controls	OR	95% CI	*P*-value
**COX-2 -765 C>G (rs20417)**						
C/C + C/G*	+	24	9	10.133	3.598–28.539	<0.001
G/G*	+	40	26	5.846	2.489–13.730	<0.001
C/C + C/G	-	11	22	1.9	0.696–5.188	0.210
G/G	-	10	38	Reference		
**IOR**				0.912	0.235–3.541	0.894
**COX-2 -1195 A>G (rs689466)**						
A/A*	+	40	11	18.182	6.359–51.989	<0.001
G/A + G/G*	+	24	24	5	1.859–13.446	0.001
A/A	-	14	25	2.8	0.987–7.940	0.053
G/A + G/G	-	7	35	Reference		
**IOR**				1.299	0.333–5.064	0.707
**EGF +61 A>G (rs4444903)**						
A/A + G/A*	+	46	24	19.167	4.129–88.963	<0.001
G/G*	+	18	11	16.363	3.188–83.993	0.001
A/A + G/A	-	19	40	4.75	1.005–22.441	0.049
G/G	-	2	20	Reference		
**IOR**				0.247	0.041–1.482	0.126
**EGFR R521K G>A (rs2227983)**						
G/G*	+	30	21	4.285	1.677–10.958	0.002
G/A + A/A*	+	34	14	7.286	2.740–19.374	<0.001
G/G	-	12	33	1.091	0.400–2.974	0.865
G/A + A/A	-	9	27	Reference		
**IOR**				0.539	0.146–1.989	0.354
**Tumor suppressor P53 P72R C>G (rs1042522)**						
C/C + C/G*	+	32	10	10.4	3.980–27.179	<0.001
G/G*	+	32	25	4.16	1.810–9.560	0.001
C/C + C/G	-	9	21	1.393	0.505–3.840	0.522
G/G	-	12	39	Reference		
**IOR**				1.795	0.468–6.882	0.394

Risk of LSCC is expressed as Crude OR. (*) Indicates a statistically significant association between risk factors and LSCC risk.

Laryngeal cancer OR is highly increased by the joint effect of genotype and cigarette consumption, with highest effect attributed to the COX2 c.-1329A>G SNP (OR: 18.18, 95% CI: 6.359–51.989, *P*-value <0.001), followed by the effect of TP53 (P72R) c.215C>G SNP (OR: 10.4; 95% CI: 3.980–27.179, *P*-value <0.001), and COX2 c.-899G>C (OR: 10.133, 95% CI: 3.598–28.539, *P*-value <0.001). However, the resulting IOR for these SNPs reveal a possible synergic relation between cigarette smoking and the genetic risk factor only for the former two SNPs, none of which were statistically significant (IOR: 1.299, 95% CI: 0.333–5.064, *P*-value: 0.707 for COX2 c.-1329A>G SNP; IOR: 1.795, 95% CI: 0.468–6.882, *P*-value: 0.394 for TP53 c.215C>G(P72R) SNP; IOR: 0.912, 95% CI: 0.235–3.541, *P*-value: 0.894 for COX2 c.-899G>C). For the EGF c.-382A>G SNP, the risk was significantly increased when the A/- genotypes (A/A plus A/G) were combined to smoking habit (OR: 19.167, 95% CI: 4.129–88.963, *P*-value <0.001), but the smoking habit was also increased without the risk genotype, from 5.22 (indicated in Table 2) to 16.36 for smoking only, which indicates that there could be a confounding factor or an artifact in these results. Also, the IOR reveals no synergy effect between the genetic and environmental risk factor for EGF c.-382A>G SNP (IOR: 0.247, 95% CI: 0.041–1.482, *P*-value: 0.126). There was no observable increase in LSCC risk when EGFR c.1562G>A (R521K) risk genotype (G/G) was combined with smoking habit, but conversely, the risk seems to be reduced, without significant interaction between both factors (IOR: 0.539, 95% CI: 0.146–1.989, *P*-value: 0.354). These results suggest a possible synergy between the genes and cigarette consumption in risk of LSCC for COX2 c.-1329A>G, and TP53 c.215C>G (P72R) SNPs which needs to be confirmed, and no synergic interaction between COX2 c.-899G>C, EGF c.-382A>G and EGFR c.1562G>A (R521K) SNPs.

## Discussion

In the present study, we determine the frequencies of COX2 c.-899G>C, COX2 c.-1329A>G, EGF c.-382A>G, EGFR c.1562G>A (R521K), and TP53 c.215C>G (P72R) SNPs in a sample of Chilean population of the city of Santiago, and demonstrate that these polymorphisms, affecting genes related to inflammation, and tumor suppression can modify the risk of LSCC.

The COX2 c.-1329A>G polymorphism had a higher impact in LSCC risk among the studied population, considering a recessive penetrance model, with an AOR of 3.27. Chronic inflammation is one of the key processes associated with carcinogenesis, and COX2 is a central enzyme involved in inflammatory signaling cascades. COX2 catalyzes the synthesis of Prostaglandin H2, the precursor molecule of all prostaglandins, including Prostaglandin E2, the main prostaglandin associated with carcinogenic inflammation. PGH2 synthesis, mediated mainly by COX2, is the limiting step in the pathogenic proinflammatory signaling cascade. Therefore, a higher COX2 expression could lead to a higher concentration of prostaglandins, contributing to malignant transformation of the tissue. The COX2 c.-1329A>G polymorphism is located in the promoter region of the gene sequence, and the A allele creates a binding sequence for the c-MYB transcription factor, which is absent in the promoter region coding for the G allele. The presence of the c-MYB regulatory element in the promoter region of the COX2 gene induces significant overexpression of COX2 mRNA in experimental models, which can contribute to an increased inflammatory environment, invasiveness, and angiogenesis mediated by prostaglandin signaling in the epithelial tissue of the larynx, a constantly challenged tissue susceptible to pro-inflammatory cues [12,13]. We also found a protector effect for the heterozygous genotype for this SNP when analyzing the data using a different penetrance model of co-dominance, which we attribute to an artifact in the results attributable to the small sample size, especially if we consider that the recessive model has better significance compared with the co-dominant model for the analyzed data, and according to this, two copies of the risk allele are needed to observe a pathogenic effect in the phenotype.

The second polymorphism with a significant association to LSCC is the TP53 c.215C>G (P72R) SNP, where the C allele was associated with increased risk with an AOR of 1.94, considering a dominant penetrance model. The protein P53 has a critical role in genomic integrity maintenance through three main mechanisms: induces cell cycle arrest, promotes DNA repair processes, and induces apoptosis in case of irreparable damage in the cell. This specific SNP is not associated with protein dysfunction, compared with other variants that impair functional domains of the protein and are directly associated with hereditary cancer, as in Li-Fraumeni’s syndrome. The C allele produces a protein with a Proline amino acid in codon 72, which has been associated with a lower tendency to induce apoptosis in transformed cells and tends more likely to induce cell cycle arrest, in comparison with p53 Arg 72 (G allele) which has better pro-apoptotic activity, according to the results described by Pim and Banks (2004) [22]. This phenomenon could explain why carriers of the C allele have an increased risk of developing LSCC according to our results [22,23].

The fact that COX2 c.-899G>C, EGF c.382A>G, and EGFR c.1562G>A polymorphisms were not directly associated with cancer risk is not surprising, considering that most of these polymorphic variants are low penetrance risk factors that have a minor influence in protein expression or function. Although the situation changes when the risk genotypes are combined with tobacco smoking habit, which increases LSCC risk for COX2 c.-899G>C, COX2 c.-1329A>G, TP53 c.215C>G (P72R), and EGF c.-382A>G SNPs, this synergy between the environmental and genetic risk factors was not significant according to the resulting IORs. Despite these results, we cannot discard the possibility of a synergic interaction between cigarette smoking and COX2 c.-1329A>G or TP53 c.215C>G SNP, where a clear tendency is observed with an IOR higher than 1, which need to be confirmed or discarded in the future with a bigger sample size, needed to detect risk factor interactions with an appropriate statistical detection power.

In prior publications, cross-talk between COX2 and EGFR signaling pathways have been described: EGF/EGFR signaling has been associated with increased COX2 expression, and COX2 bioproduct Prostaglandin E2 activity have also been linked to EGFR transactivation, which could result in positive feedback and potentiation between these signaling cascades [10,32]. This phenomenon, combined with a reduced pro-apoptotic activity of the P53 72Pro protein, could lead to an increased proliferative/mutagenic potential, and therefore, increased LSCC risk [23]. These hypothesized biologic relationships need to be confirmed by animal model or cell culture experiments, in order to observe the direct influence and joint interactions between the studied genes, proteins, and SNPs under controlled experimental conditions.

The present study has several limitations that need to be considered when interpreting the observed results. One of these limitations is the small sample size, which increases the error rate, observed as the large confidence intervals of the presented results. This limitation could be corrected in the future with increased sample size. However, it is remarkable that, despite the small sample, strong associations and statistically significant results were obtained in the present study, which could be attributed to a strong biological impact of the genes observed in this research results.

The most remarkable finding of the present study is how the effect of the COX2 c.-1329A>G, and TP53 c.215C>G (P72R) SNPs risk alleles result in a dramatic impact in LSCC risk within the studied population. The next step would be to confirm the biological interactions between the studied SNPs through *in-vivo* or *in-vitro* experiments, to define the precise mechanism of enhanced tumorigenicity, confirm the impact of joint interaction of the studied polymorphic variants in tumor growth and malignant transformation, and collect more evidence that supports a possible synergy between these SNPs.

The knowledge provided by the present study could bring novel biomarkers of LSCC and other Head and Neck squamous cell carcinomas, and through exploration of the mechanisms of malignant transformation described in this research, novel therapeutic targets for LSCC, like COX2 inhibitors combined to already existing EGFR antibodies, could be developed in the future as strategies for treating this type of cancer.

## Supplementary Material

Supplementary Figure S1

## Data Availability

The datasets used and/or analyzed in the present study are available on reasonable request to the corresponding authors.

## Abbreviations

AOR: adjusted odds ratio
COX2: cyclooxygenase-2
EGF: epidermal growth factor
EGFR: EGF receptor
GCP: good clinical practices
IOR: interaction odds ratio
LSCC: laryngeal squamous cell carcinoma
OR: odds ratio
PCR: polymerase chain reaction
RFLP: Restriction Fragment Length Polymorphism
SNP: single nucleotide polymorphism

## References

[1] American Cancer Society (2018) Cancer Facts & Figures 2018, American Cancer Society, Atlanta, GA, http://www.cancer.org/content/dam/CRC/PDF/Public/8664.00.pdf

[2] Marur S. and Forastiere A.A. (2008) Head and neck cancer: changing epidemiology, diagnosis, and treatment. Mayo Clin. Proc. 83, 489–501 10.4065/83.4.489 18380996

[3] Barnes L., Eveson J.W., Reichart P. and Sidransky D. (2005) Tumours of the hypopharynx, larynx and trachea. In Pathology and Genetics of Head, pp. 108–162, IARCPress, Lyon

[4] Maasland D.H., van den Brandt P.A., Kremer B., Goldbohm R.A. and Schouten L.J. (2014) Alcohol consumption, cigarette smoking and the risk of subtypes of head-neck cancer: results from the Netherlands Cohort Study. BMC Cancer 14, 187 10.1186/1471-2407-14-187 24629046 PMC4004328

[5] Hecht S. (1999) Tobacco smoke carcinogens lung cancer. J. Natl. Cancer Inst. 91, 1194–1210 10.1093/jnci/91.14.1194 10413421

[6] Seitz H. and Stickel F. (2007) Molecular mechanisms of alcohol mediated carcinogenesis. Nat. Rev. Cancer 7, 599–612 10.1038/nrc2191 17646865

[7] Masood N., Yasmin A. and Kayani M.A. (2014) Genetic variations and head and neck cancer risks. Mol. Biol. Rep. 41, 2667–2670 10.1007/s11033-014-3125-6 24452716

[8] Maurya S.S., Anand G., Dhawan A., Khan A.J., Jain S.K., Pant M.C. et al. (2014) Polymorphisms in drug-metabolizing enzymes and risk to head and neck cancer: evidence for gene-gene and gene-environment interaction. Environ. Mol. Mutagen. 55, 134–144 10.1002/em.21837 24519899

[9] Bakhle Y.S. (2001) COX-2 and cancer: a new approach to an old problem. Br. J. Pharmacol. 134, 1137–1150 10.1038/sj.bjp.0704365 11704632 PMC1573056

[10] Gasparini G., Longo R., Sarmiento R. and Morabito A. (2001) Inhibitors of cyclo-oxygenase 2: a new class of anticancer agents? Lancet Oncol. 4, 605–615 10.1016/S1470-2045(03)01220-8 14554238

[11] Campanholo V.M.L.P., Felipe A.V., Lima J.M., Pimenta C.A.M., Ventura R.M. and Forones N.M. (2014) -765 G>C Polymorphism of the COX-2 gene and gastric cancer risk in Brazilian population. Arq. Gastroenterol. 51, 79–83 25003256 10.1590/s0004-28032014000200002

[12] Zhang X., Lin D., Miao X., Tan W., Ning B., Liu Z. et al. (2005) Identification of functional genetic variants in cyclooxygenase-2 and their association with risk of esophageal cancer. Gastroenterology 129, 565–576 16083713 10.1016/j.gastro.2005.05.003

[13] Shin W.G., Kim H.J., Cho S.J., Kim H.S., Kim K.G., Jang M.K. et al. (2012) The COX-2-1195AA genotype is associated with diffuse-type gastric cancer in Korea. Gut Liver 6, 321–327 10.5009/gnl.2012.6.3.321 22844559 PMC3404168

[14] Upadhyay R., Jain M., Kumar S., Ghoshal U.C. and Mittal B. (2009) Functional polymorphisms of cyclooxygenase-2 (COX-2) gene and risk for esophageal squamous cell carcinoma. Mutat. Res. 663, 52–59 10.1016/j.mrfmmm.2009.01.007 19428370

[15] Gullick W.J. (1991) Prevalence of aberrant expression of the epidermal growth factor receptor in human cancers. Br. Med. Bull. 47, 87–98 10.1093/oxfordjournals.bmb.a072464 1863851

[16] Zeng F. and Harris R.C. (2014) Epidermal growth factor, from gene organization to bedside. Semin. Cell Dev. Biol. 28, 2–11 10.1016/j.semcdb.2014.01.011 24513230 PMC4037350

[17] Wang W.Z., Liu Q., Sun J.J., Zuo W.S., Hu D.W., Ma S.G. et al. (2015) Correlation between P53 and epidermal growth factor receptor expression in breast. Genet. Mol. Res. 14, 4282–4290 10.4238/2015.April.28.10 25966200

[18] Shahbazi M., Pravica V., Nasreen N., Fakhoury H., Fryer A.A., Strange R.C. et al. (2002) Association between functional polymorphism in EGF gene and malignant melanoma. Lancet 359, 397–401 10.1016/S0140-6736(02)07600-6 11844511

[19] Araújo A., Costa B., Pinto-Correia A., Fragoso M., Ferreira P., Dinis-Ribeiro M. et al. (2011) Association between EGF +61A/G polymorphism and gastric cancer in Caucasians. World J. Gastroenterol. 17, 488–492 10.3748/wjg.v17.i4.488 21274378 PMC3027015

[20] Su N.W., Leu Y.S., Lee J.C., Liu C.J., Cheng C.Y., Lin J.S. et al. (2014) EGF and EGFR genetic polymorphisms predict prognosis in locally advanced pharyngolaryngeal squamous cell carcinoma patients receiving postoperative concurrent chemoradiotherapy. Onco Targets Ther. 7, 2197–2204 10.2147/OTT.S70188 25506224 PMC4259259

[21] Mustafa O.H., Hamzeh A.R., Ghabreau L., Akil N., Almoustafa A.E. and Alachkar A. (2013) Allele frequencies of the epidermal growth factor receptors polymorphism R521K in colorectal cancer patients and healthy subjects indicate a risk-reducing effect of K521 in Syrian population. N. Am. J. Med. Sci. 5, 202–206 23626956 10.4103/1947-2714.109189 PMC3632024

[22] Pim D. and Banks L. (2004) P53 polymorphic variants at codon 72 exert different effects on cell cycle progression. Int. J. Cancer 108, 196–199 10.1002/ijc.11548 14639602

[23] Thomas M., Kalita A., Labrecque S., Pim D., Banks L. and Matlashewski G. (1999) Two polymorphic variants of wild-type P53 differ biochemically and biologically. Mol. Cell. Biol. 19, 1092–1100 10.1128/MCB.19.2.1092 9891044 PMC116039

[24] Cattelani S., Ferrari-Amorotti G., Galavotti S., Defferrari R., Tanno B., Cialfi S. et al. (2012) The P53 codon 72 Pro/Pro genotype identifies poor-prognosis neuroblastoma patients: correlation with reduced apoptosis and enhanced senescence by the P53-72P isoform. Neoplasia 14, 634–643 10.1593/neo.12594 22904680 PMC3421959

[25] Irarrázabal C., Rojas C., Orellana C., Acevedo C., Huidobro C., Cáceres D. et al. (2006) Joint effect among P53, CYP1A1, GSTM1 polymorphism combinations and smoking on prostate cancer risk: an exploratory genotype-environment interaction study. Asian J. Androl. 8, 349–355 16625286 10.1111/j.1745-7262.2006.00135.x

[26] Loeb K.R., Asgari M.M., Hawes S.E., Feng Q., Stern J.E., Jiang M. et al. (2012) Analysis of Tp53 codon 72 polymorphisms, Tp53 mutations, and HPV infection in cutaneous squamous cell carcinomas. PLoS ONE 7, e34422 10.1371/journal.pone.0034422 22545084 PMC3335843

[27] Shahbazi M., Pravica V., Nasreen N., Fakhoury H., Fryer A.A., Strange R.C. et al. (2002) Association between functional polymorphism in EGF gene and malignant melanoma. Lancet 359, 397–401 10.1016/S0140-6736(02)07600-6 11844511

[28] Sasaki H., Okuda K., Shimizu S., Takada M., Kawahara M., Kitahara N. et al. (2009) EGFR R497K polymorphism is a favorable prognostic factor for advanced lung cancer. J. Cancer Res. Clin. Oncol. 135, 313–8 10.1007/s00432-008-0464-5 18726117 PMC12160300

[29] Ueda M., Hung Y.C., Terai Y., Saito J., Nunobiki O., Noda S. et al. (2005) Glutathione-S-transferase and p53 polymorphisms in cervical carcinogenesis. Gynecol. Oncol. 96, 736–740 10.1016/j.ygyno.2004.11.005 15721419

[30] Botto L.D. and Khoury M.J. (2001) Commentary: facing the challenge of gene-environment interaction: the two-by-four table and beyond. Am. J. Epidemiol. 153, 1016–1020 10.1093/aje/153.10.1016 11384958

[31] Yang Q. and Khoury M.J. (1997) Evolving methods in genetic epidemiology. III. Gene–environment interaction in epidemiologic research. Epidemiol. Rev. 19, 33–43 10.1093/oxfordjournals.epirev.a017944 9360900

[32] Pai R., Soreghan B., Szabo I.L., Pavelka M., Baatar D. and Tarnawski A.S. (2002) Prostaglandin E2 transactivates EGF-receptor: a novel mechanism for promoting colon cancer growth and gastrointestinal hypertrophy. Nat. Med. 8, 289–293 10.1038/nm0302-289 11875501

