# Supplementary material for: Genetic polymorphisms as non-modifiable susceptibility factors to laryngeal cancer
Source: Biosci Rep. 2020 May 5;40(5):BSR20191188. doi: 10.1042/BSR20191188 (PMC7201556; doi:10.1042/BSR20191188)
Supplement: Supplementary Figure S1 [file BSR-2019-1188_supp.pdf]

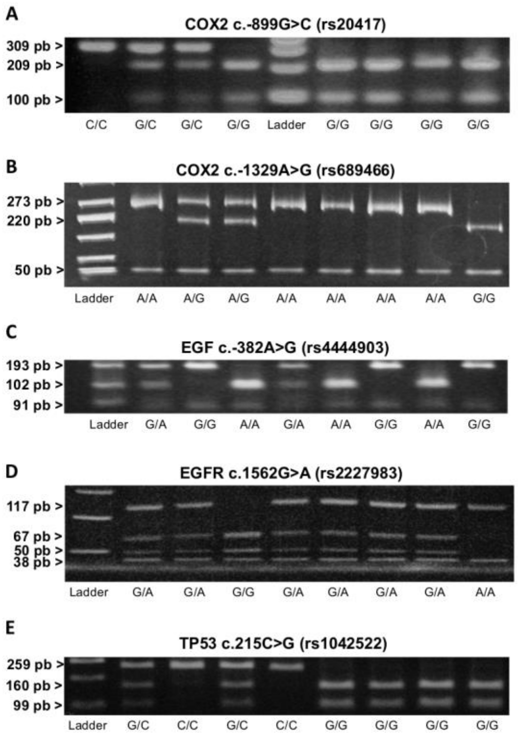
A
COX2 c.-899G>C (rs20417)
309 pb >
209 pb >
100 pb >
C/C G/C G/C G/G Ladder G/G G/G G/G G/G
B
COX2 c.-1329A>G (rs689466)
273 pb >
220 pb >
50 pb >
Ladder A/A A/G A/G A/A A/A A/A A/A G/G
C
EGF c.-382A>G (rs4444903)
193 pb >
102 pb >
91 pb >
Ladder G/A G/G A/A G/A A/A G/G A/A G/G
D
EGFR c.1562G>A (rs2227983)
117 pb >
67 pb >
50 pb >
38 pb >
Ladder G/A G/A G/G G/A G/A G/A G/A A/A
E
TP53 c.215C>G (rs1042522)
259 pb >
160 pb >
99 pb >
Ladder G/C C/C G/C C/C G/G G/G G/G G/G

Supplementary Figure 1: Representative profile of genotype analyses of selected subjects. a) COX2 c.-899G>C (rs20417) genotypes; b) COX2 c.-1329 A>G (rs689466) genotypes; c) EGF c.-382A>G (rs4444903) genotypes; d) EGFR c.1562G>A (rs2227983) genotypes and e) TP53 c.215C>G (rs1042512) genotypes.
